# Potential ecotoxicological effects of silver nanoparticles and silver sulphide on the endogeic earthworm *Aporrectodea caliginosa* (Savigny 1826)

**DOI:** 10.1007/s10646-023-02705-z

**Published:** 2023-10-20

**Authors:** Jeannette M. Kister, Christopher N. Lowe, Kevin R. Butt

**Affiliations:** Natural Sciences, University of Central Lancashire, Preston, PR1 2HE UK

**Keywords:** Silver nanoparticles, *Aporrectodea caliginosa*, Silver sulphide, Ecotoxicology, Avoidance, Reproduction

## Abstract

Silver nanoparticles (AgNPs) are increasingly used in consumer products and subsequently arrive in wastewater systems, accumulating as silver sulphide (Ag_2_S) in the resulting biosolids, which are commonly spread onto agricultural fields as a fertiliser. Experiments were performed to investigate the effect of AgNPs, using the endogeic earthworm *Aporrectodea caliginosa* as a test organism. In an acute toxicity experiment, *A. caliginosa* were exposed to soil containing different concentrations of AgNPs (0, 1, 5, 10, 50, 100, 250, 500, 750, and 1000 mg kg^−1^ dry soil) and Ag_2_S (0, 10, 50, 100, 500, and 1000 mg kg^−1^ dry soil). Earthworm biomass and mortality were monitored. Earthworms exposed to 500, 750 and 1000 mg kg^−1^ fresh AgNPs had mortality rates of 20%, 60% and 70%, respectively. Changes in biomass were directly related to AgNP concentration. Exposure to Ag_2_S did not affect biomass or mortality. Further experiments used 0, 10, 50, 100 and 250 mg kg^−1^ AgNPs and 0, 50, 100, 500, and 1000 mg kg^−1^ Ag_2_S to evaluate sublethal effects on *A. caliginosa*. Avoidance behaviour in a linear gradient was evaluated after 14 days. Earthworms significantly preferred soil that was free of either AgNPs or Ag_2_S. The same concentrations were used to assess effects on cocoon production of *A. caliginosa* exposed to AgNPs and Ag_2_S. In the first 3 months of AgNP exposure, higher concentrations had a negative effect on cocoon production, but this effect diminished thereafter. Ag_2_S had no discernible effect on reproduction. Overall, introduction of AgNPs into the soil through the application of biosolids appears to be of low concern to the tested endogeic earthworm.

## Highlights

- High concentrations of AgNPs are toxic towards *A. caliginosa.*
- Earthworm mass affects susceptibility towards AgNP-toxicity.
- *Aporrectodea caliginosa* avoids low concentrations of AgNPs.
- Silver sulphide is less toxic *to A. caliginosa* than AgNPs.

## Introduction

The antimicrobial properties of silver have been known for centuries and by the manufacture of nanoparticles from it, the range of possible applications has been expanded. Consequently, the occurrence of silver nanoparticles (AgNPs) in consumer products has also increased (Nowack et al. 2011). From clothing, cleaning supplies, medical equipment to cosmetics, AgNPs are ubiquitous. However, with use, the AgNPs tend to be washed away and reach the wastewater system, where they amass in biosolids (treated sewage sludge) and may ultimately reach the soil as in the UK, 80% of those biosolids are applied to fields (DEFRA 2012). Another way of silver reaching agricultural systems is the potential for AgNPs to be utilised as an antifungal product sprayed directly on to plants (Jo et al. 2009; Lamsa et al. 2011). As a result of this increased domestic use, in addition to their commercial applications, AgNPs have been widely studied to include their effects on aspects of the environment.

Nevertheless, the likelihood of pristine AgNPs reaching the soil through the application of biosolids to agricultural fields is low. Studies have shown that AgNPs react with different forms of sulphur in the sewage treatment process and are transformed to silver sulphide (Ag_2_S) (Kim et al. 2010; Doolette et al. 2013). Ag_2_S is nearly insoluble in water and has been shown to have a lower bioavailability than AgNPs, for example in experiments with the nematode *Caenorhabditis elegans* (Starnes et al. 2015).

Within soil investigations and analyses, earthworms are a widely recommended group of organisms, used to assess toxicity, with common acute endpoints such as survival or changes in mass, but others include cocoon production and avoidance behaviour (OECD 1984; OECD 2016). Avoidance and reproduction are more sensitive endpoints and can show the effects of sub-acute exposure to toxic substances in the soil (Lowe and Butt 2007). All these tests recommend the use of epigeic earthworm species (*sensu* Bouché 1977) which live and feed above mineral soil, although it might be preferable to use endogeic earthworms to perform ecotoxicological testing, as these live and feed in the soil and are therefore more affected by any soil contamination (Bart et al. 2018). Multiple studies have been performed on the effects of AgNPs on earthworms, utilising predominantly epigeic species, and have been shown to be toxic to varying degrees (Heckmann et al. 2011; Shoults-Wilson et al. 2011c; Choi and Park 2015). It is suggested that the toxicity of AgNPs is due to the slow release of Ag^+^ ions (Shoults-Wilson et al. 2011a; Brami et al. 2017).

The overarching aim of this work was to investigate potential ecotoxicological effects of silver nanoparticles and silver sulphide on an endogeic earthworm. Specific objectives were to determine the effects of uncoated AgNPs and Ag_2_S on the survival, biomass, reproduction, and avoidance behaviour of *Aporrectodea caliginosa*. This earthworm was chosen as it is commonly found in agroecosystems and therefore directly impacted by soil contamination. We hypothesised that Ag_2_S would be less toxic towards *A. caliginosa* due to a lower bioavailability and lower potential to release Ag^+^ ions.

## Materials and methods

All work was undertaken in laboratories at the University of Central Lancashire (UCLan). As close as possible, and where applicable, techniques followed those previously undertaken with similar experimentation (e.g., Lowe and Butt 2005; Brami et al. 2017). Information provided below first describes the materials utilised and then the specific experimental approaches to address the given objectives.

### (Nano)Particles

Uncoated 80 nm AgNPs were purchased online in the form of a nano-powder (GetNanoMaterials.com), whereas Ag_2_S particles were synthesised at UCLan according to the supplementary information provided by Sadovnikov et al. (2016). For this synthesis, masses of 8.49 g of AgNO_3_ and 12.9 g of citrate dihydrate were dissolved in 100 mL Millipore water (ultrapure water); with 6.5 g of sodium sulphide hydrate (60%) dissolved separately in 100 mL Millipore water. After preparation of both solutions, they were combined in a 250 mL Duran bottle which was vortexed until the previously white solution turned black. The solution was then placed in an ultrasound bath (Grant Ultrasonic 3 L Bath) for 30 mins, and kept in the dark at ambient temperature for 3 days, sonicated again for 20 mins and washed with Millipore water before the particles were airdried (Sadovnikov et al. 2016). Particles were analysed using a Thermo Scientific Quattro S scanning electron microscope (SEM) with Energy Dispersive X-Ray Analysis (EDX). The SEM was used to determine particle size and EDX was used to determine elemental composition.

### Earthworms

Adults of the endogeic earthworm *Aporrectodea caliginosa*, identified using Sims and Gerard (1999), were collected by manual extraction (digging and hand-sorting of soil) from pasture at Bottom’s Farm, Preston, Lancashire and acclimated to laboratory conditions. Before use in AgNP acute toxicity experimentation, earthworms were kept for 4 weeks in Kettering loam, fed with horse manure in a temperature-controlled incubator (LMS, Kent) at 15 ^o^C, with a 24 h dark cycle (Lowe and Butt 2005; Fründ et al. 2010). All horse manure was locally sourced from un-wormed animals, and kept frozen at −4 °C until thawed and dried at 120 °C for 24 h. For all other investigations, laboratory-reared *A. caliginosa* were used. These were the offspring of the collected earthworms.

### Earthworm survival and change in biomass

In two separate experiments, *A. caliginosa* were exposed to either AgNPs or Ag_2_S for at least 14 days, to determine effects on earthworm survival and potential change in biomass.

These acute toxicity experiments were based on previous work by Brami et al. (2017), with *A. caliginosa* replacing *Allolobophora chlorotica* (another endogeic species). The experimental medium used was Kettering Loam (Boughton Loam Company), a sterilised soil. To this, 10% dried and milled (<2 mm) horse manure (HM) and 5% sand (Hanson kiln dried sand (lime-free washed silica, average grain size 0.5 mm)) was added. All dry ingredients were mixed thoroughly and then wetted to approximately 25–30% moisture with tap water. The sand was a vehicle to introduce AgNPs and Ag_2_S into the soil (Brami et al. 2017). Prior to addition, the particles were mixed into the sand. Final treatment concentrations chosen for these experiments were 0, 1, 10, 100, 250, 500 and 1000 mg AgNPs kg^−1^ soil and 0, 10, 50, 100, 500, and 1000 mg Ag_2_S kg^−1^ soil. For each treatment, five food grade 250 mL circular plastic tubs (11.5 cm dia., from cater4you Ltd.) were filled with the given medium and two healthy (no obvious morphological defects), adult (presence of swollen clitellum) *A. caliginosa* were added. Each earthworm had biomass and general condition recorded prior to the experiment, after 7 days and at experimental end (14 days). Due to no observable changes in biomass and survival of all earthworms, the Ag_2_S experiment was extended to 28 days with sampling every 7 days.

### Reproduction

In two separate experiments, *A. caliginosa* were exposed to AgNPs and Ag_2_S for 4 months to assess and compare the potential impact of the particles on endogeic earthworm reproduction, another recommended method for assessing soil toxicity (OECD 2016).

Soil used in the reproduction experiment consisted of Kettering Loam with 50–100 g kg^−1^ HM (dried, milled and sieved <2 mm) and 5 g kg^−1^ sand as a vehicle for AgNPs and Ag_2_S, or unadulterated in a control medium. HM concentration was 50 g kg^−1^. Cocoon production and change in biomass was recorded for 30 pairs of *A. caliginosa*. This was initially under 4 weeks of control conditions before the start of experiments to ensure reproductively active animals were used. Based on these results, 5 experimental groups were established, all with comparable earthworm mass and cocoon production ability.

Based on results from survival experiments (Section “Earthworm survival and change in biomass”) treatment concentrations used here were 0, 10, 50, 100, and 250 mg kg^−1^ for AgNPs and 0, 50, 100, 500, and 1000 mg kg^−1^ for Ag_2_S. Earthworms were again housed in 250 mL plastic tubs and kept in an incubator at 15 °C in darkness (Lowe and Butt 2005). Cocoon production and earthworm biomass were assessed every 4 weeks for a total of 20 weeks. In the Ag_2_S experiment, one week of sampling was inadvertently delayed, which resulted in sampling points at 4, 8, 13, 17, and 21 weeks.

### Avoidance behaviour

In separate experiments, avoidance behaviour was assessed following the methodology of Lowe et al. (2016), to assess effects of either AgNPs or Ag_2_S on *A. caliginosa*, below previously determined levels of acute or reproductive toxicity.

Linear avoidance chambers were used, which consisted of long plastic planter troughs (0.6 m × 0.13 m × 0.1 m) with engineered, removable dividers, to create 5 equally spaced sections, each containing approximately 1.4 L of substrate (position marked on the edge of the trough). Five replicate chambers were used. Experimental treatments in the sections of each chamber were 0, 10, 50, 100, and 250 mg kg^−1^ for AgNP- and 0, 50, 100, 500, and 1000 mg kg^−1^ for Ag_2_S-containing soils, with the addition of 10 g kg^−1^ HM. In each experiment, treatment concentrations formed a linear gradient from control at one end, with adjacent increasing concentrations to the highest treatment at the other end. Once constructed, a single earthworm was placed on to the surface of each section in each replicated chamber. Once all earthworms had burrowed into the soil, the dividers were removed, a slight shake applied to ensure the soil formed a continuum, and the avoidance chambers were sealed tight with plastic film. After addition of small holes into the film to allow air circulation, the chambers were placed into an incubator at 15 °C in darkness for 14 days.

At the end of the experiment, each chamber was sampled separately. After careful removal of the plastic film to avoid disturbance, the dividers were forcibly re-inserted into the soil at the original marked positions. Thereafter, the soil from each section was removed and hand-sorted for earthworms. Each earthworm located had its position recorded and mass determined. Any earthworms cut into two parts by divider insertion were counted as 0.5 earthworms for the 2 given sections.

### Statistical analysis

Statistical analysis was performed using the software SPSS (Version 28). One-way analysis of variance (ANOVA) was used to analyse data, with a Tukey post-hoc test where appropriate, or with a Fisher-LSD. A value of *p* < 0.05 was considered to indicate statistical significance. All results are expressed as mean ± standard deviation.

## Results

### Earthworm survival and change in biomass

#### AgNPs

At all treatment levels tested, adult *A. caliginosa* survived to 7 days. After 14 days, 20, 60 and 70% mortality were recorded at 500, 750 and 1000 mg kg^−1^ AgNP respectively. It was noticeable (Table 1) that earthworms of lower mass were more susceptible to mortality when exposed to AgNP concentrations of 500 mg kg^−1^ and higher. Table 1 is split into columns to show the mass on Day 0 of earthworms that survived the experiment and those that did not.

**Table 1:** Mean initial mass (±standard deviation) of *A. caliginosa* exposed to a range of AgNP concentrations (no earthworms died at less than 500 mg kg^−1^ AgNP) (5 replicates of 2 earthworms per trearment)

AgNP conc. in soil [mg kg^−1^]	Initial mass of surviving earthworms ±STDEV	Initial mass of dead earthworms ±STDEV
0	710.6 ± 314.2	
1	698.3 ± 325.3	
5	584.5 ± 357.4	
10	683.8 ± 293.9	
50	634.2 ± 199.0	
100	782.2 ± 325.7	
250	713.2 ± 275.0	
500	890.5 ± 258.2	272.0 ± 86
750	826.5 ± 84.6	626.2 ± 218.1
1000	1076.7 ± 61.1	677.3 ± 332.4

**Fig. 1:**
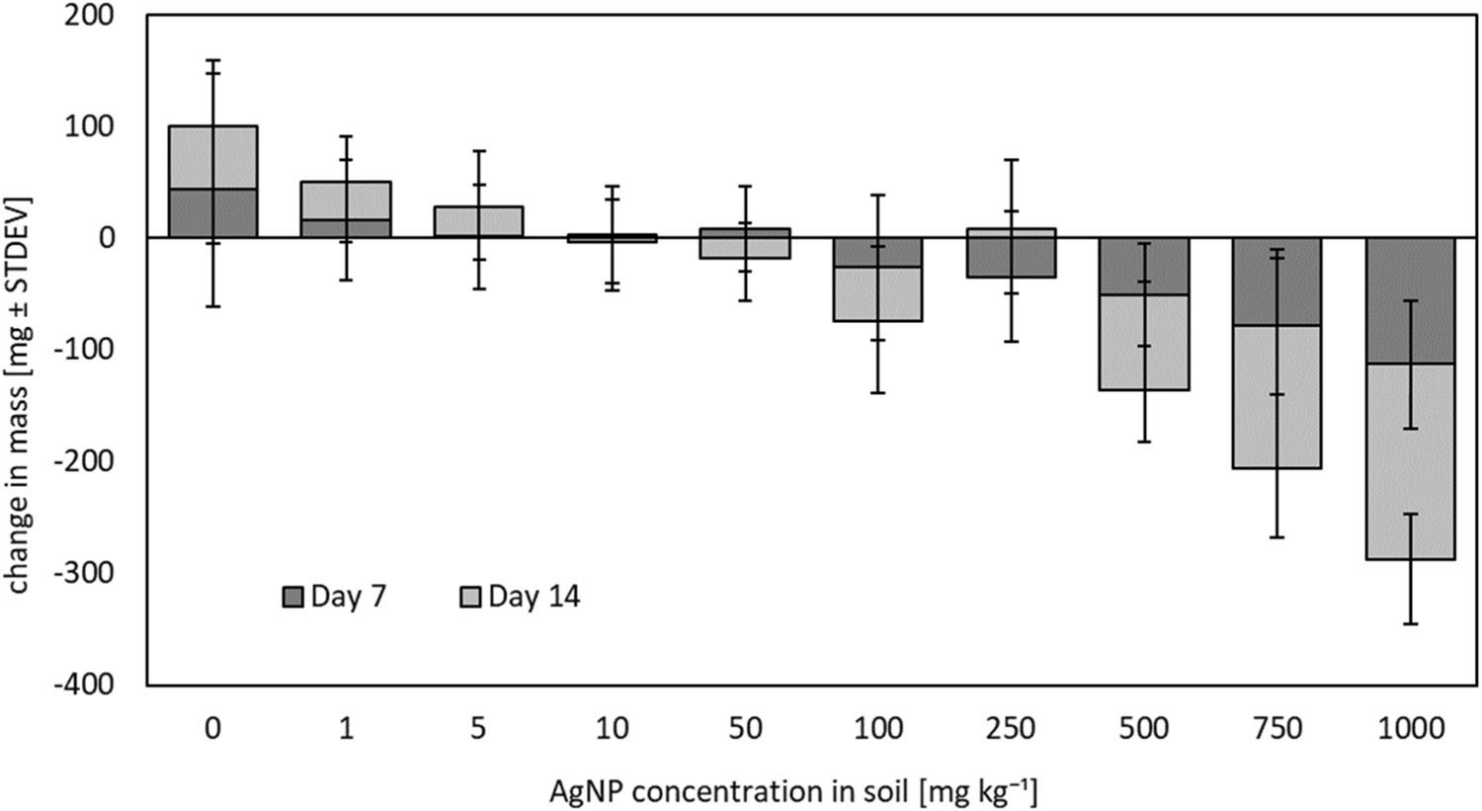
Mean change in biomass (±standard deviation) of adult *A. caliginosa* exposed to a range of AgNPs recorded after 7 and 14 days (5 replicates of 2 earthworms per trearment)

Higher soil concentrations of AgNPs caused a decrease in earthworm mass. Fig. 1 shows the cumulative change in biomass of adult earthworms exposed to fresh AgNPs, excluding masses of individuals that died. Smaller earthworms were more susceptible to mortality during the experiment, so mean mass of surviving earthworms at Day 14 was relatively similar to that at Day 0. Overall, surviving earthworms exposed to AgNP levels greater than 50 mg kg^−1^ lost body mass over the experiment.

#### Ag_2_S

**Fig. 2:**
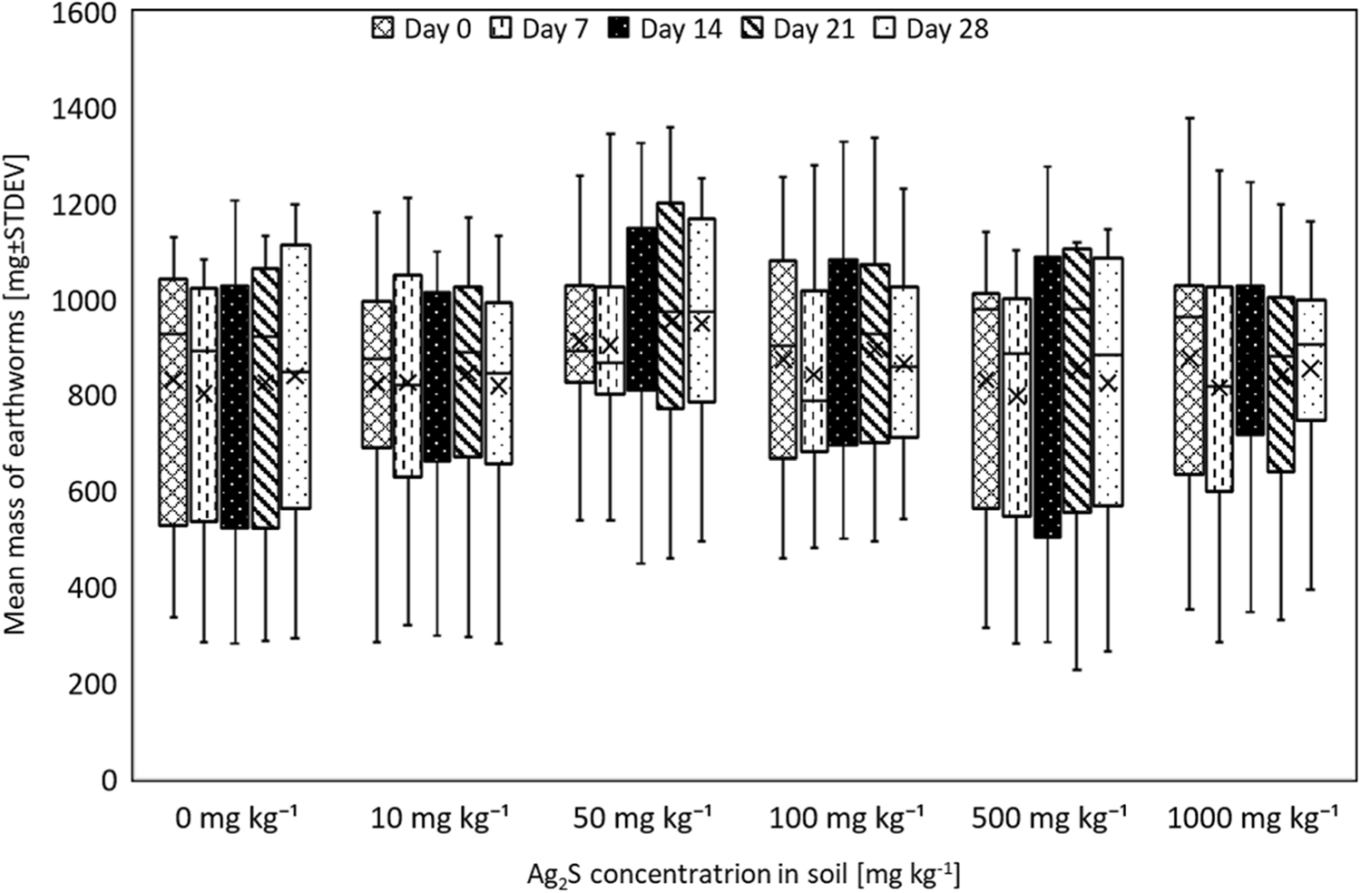
Mean biomass (±standard deviation) of adult *A. caliginosa* exposed to Ag_2_S in soil for 28 days (5 replicates of 2 earthworms per trearment)

Due to survival of all earthworms to 14 days in all treatments, this was extended to 28 days to permit observation of potentially delayed effects. All earthworms survived for the full duration of the experiment and no significant change in biomass was recorded within or between treatments with all earthworms remaining in good condition (Fig. 2).

### Reproduction

#### AgNPs

**Fig. 3:**
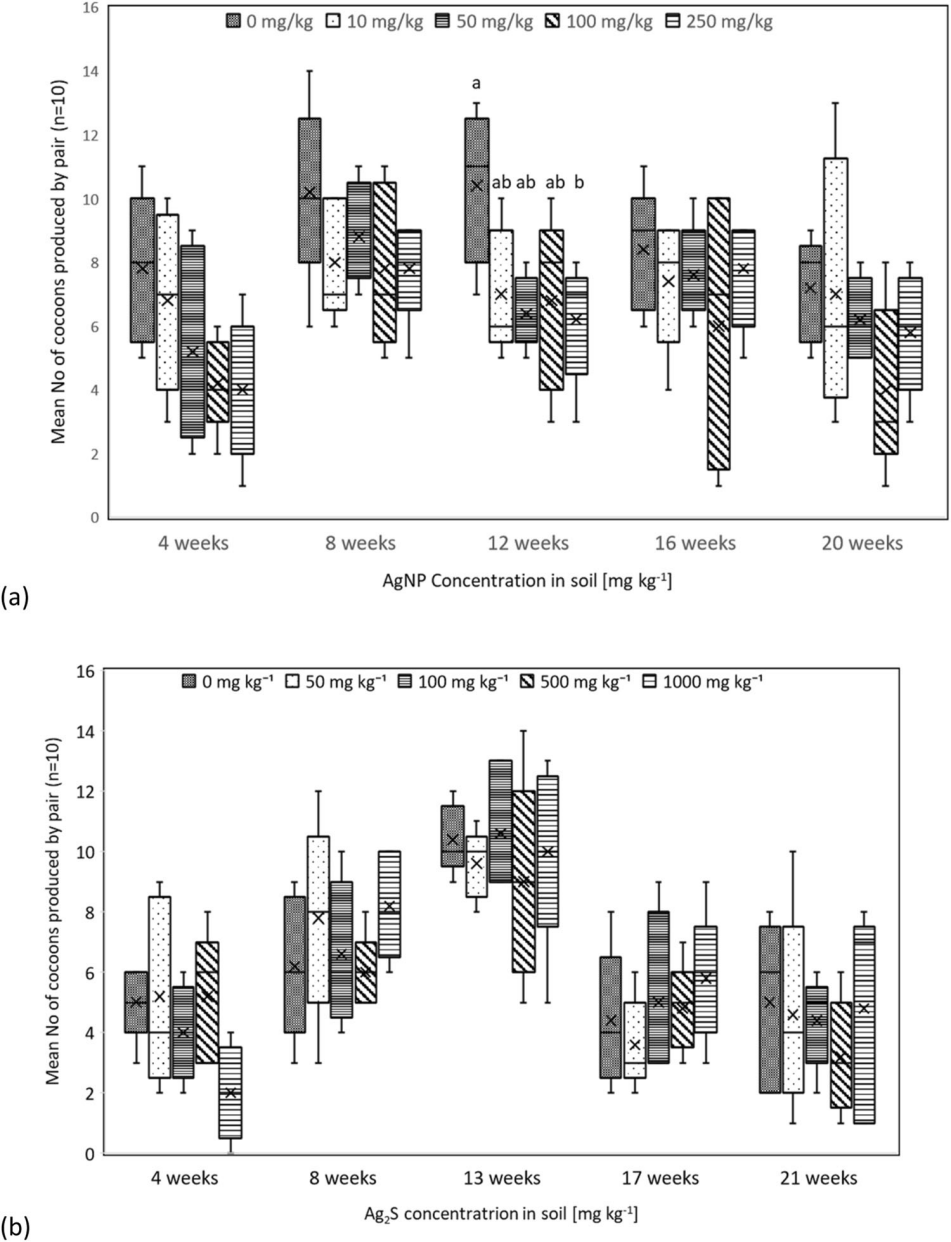
Mean number of cocoons (±standard deviation) produced by each pair of *A. caliginosa* exposed to (**a**) AgNPs and (**b**) Ag_2_S; different letters denote significant differences e.g. a is significantly different to b but not to ab (ANOVA with Tukey post-hoc, *p* < 0.05) (5 replicates of 2 earthworms per trearment)

Mean mass of *A. caliginosa* remained constant throughout. Cocoon production varied between sampling points (Fig. 3a), with a significant difference between the treatments at 12 weeks (one-way ANOVA F_(4, 20)_ = 3.320, *p* = 0.031) where *A. caliginosa* exposed to 0 mg kg^−1^ AgNPs produced significantly more cocoons than those exposed to 250 mg kg^−1^ AgNPs (Tukey post hoc: *p* = 0.038). Over the first 3 sampling points, *A. caliginosa* produced more cocoons in control soil than in soils containing AgNPs, particularly over the first 4 weeks, where a clear negative relationship between AgNP exposure and cocoons production was recorded, although this was not significant.

#### Ag_2_S

Biomass of *A. caliginosa* remained relatively constant over the duration of the experiment with no statistical differences between treatments and between sampling points (not shown). Exposure to a range of Ag_2_S levels in the soil did not affect mean *A. caliginosa* cocoon production (Fig. 3b). Earthworm pairs exposed to 1000 mg kg^−1^ had a relatively low mean cocoon production of 2 per pair (over the first 4 weeks), but differences between treatments were not significant at that point. At week 13, cocoon production increased overall, but this was due to cocoon collection from a 5-week period.

### Avoidance behaviour

#### AgNPs

**Fig. 4:**
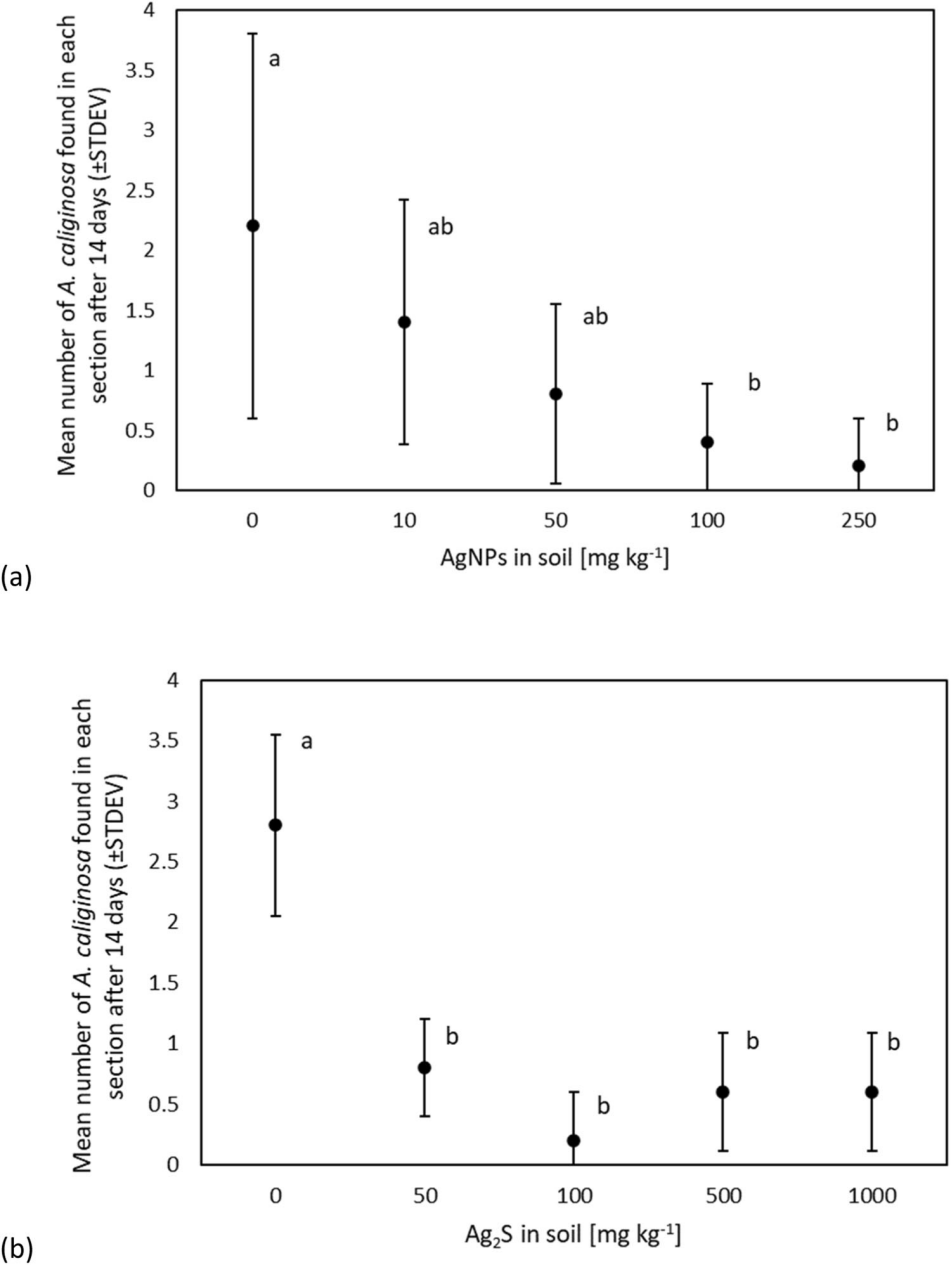
Mean (±SDEV) number of *A. caliginosa* located in different segments of an avoidance chamber with increasing levels of (**a**) AgNP and (**b**) Ag_2_S after a 14-day linear avoidance experiment (different letter denote significance in results e.g., a is significantly different to b but not to ab; ANOVA, LSD post-hoc *p* < 0.05) (5 replicate chambers, each containing 5 earthworms)

All earthworms survived the experiment and masses remained relatively constant. After 14 days, most *A. caliginosa* were in soil with no, or lower, concentrations of AgNPs (Fig. 4a). With each increase in AgNP concentration, less earthworms were found in the section of the avoidance chamber. A preference for control soil was significant (*p* = 0.048, F_(4,20)_ = 2.895) when compared with soil containing 100 and 250 mg kg^−1^ AgNP (LSD post-hoc, *p* < 0.05). There was a positive correlation (r(5) = 0.95, *p* < 0.05) between the AgNP treatment in the soil and the mean mass of earthworms found within given sections of the gradient (Table 2). Earthworms of lower mass showed a greater avoidance behaviour towards AgNP-containing soil.

**Table 2:** Mean mass (±STDEV) of *A. caliginosa* found after 14 days in the different segments of two linear avoidance chambers with soil containing AgNPs and Ag_2_S (5 replicate chambers, each containing 5 earthworms)

Concentration [mg kg^−1^]	Mean mass [mg ± STDEV]
AgNPs	
0	605.3 ± 144.4
10	529.7 ± 217.8
50	727.7 ± 203.0
100	669.5 ± 71.5
250	1138 ± 0
Ag_2_S	
0	597.57 ± 181.1
50	676 ± 64.01
100	574 ± 0
500	828.33 ± 209.05
1000	617.67 ± 48.81

#### Ag_2_S

After 14 days, the majority of *A. caliginosa* were present in the control soil and, on average, less than one earthworm was found in soil containing any amount of Ag_2_S (Fig. 4b). A one-way ANOVA showed that this result was significant (*p* < 0.001, F_(4, 20)_ = 15.588). Soil moisture content in each of the sections of the gradient was not significantly different and there was no correlation between mass of earthworms and section of the soil in which they were found (Table 2). Further, there was no observable gradient reaction, i.e., a greater avoidance of soil containing differing treatments of Ag_2_S, confirmed by Fisher LSD post-hoc analysis. Some earthworms lost mass over the course of this experiment, with the mean decreasing by 7%.

## Discussion

While new aspects relating to silver nanoparticle investigations were discovered, findings generally concur with existing literature. However, direct comparison of results is somewhat hindered by major differences between studies, such as earthworm species, soil substrate, and type specification of AgNPs such as size or coatings. AgNP toxicity is thought to be caused by Ag^+^ ion release which is affected by coatings, size, agglomeration, and oxidation (Shoults-Wilson et al. 2011a; 2011b) which corresponds with the limited toxicity of Ag_2_S towards *A. caliginosa* in this study.

### Acute

#### AgNP

AgNPs were shown to cause mortality in adult *A. caliginosa* at concentrations of 500 mg kg^−1^ and higher. This is in line with previous findings that AgNPs are toxic towards earthworms, for example *Eisenia fetida* and *A. chlorotica* (Shoults-Wilson et al. 2011a; Brami et al. 2017). However, the concentration at which AgNPs start to cause mortality differs from the results obtained by Brami et al. (2017) despite many similarities in experimental set up including the use of endogeic earthworms. The main difference is that in the 2017 study, *A. chlorotica* were used, while the current experiment used *A. caliginosa*. *Allolobophora chlorotica*, as a species, was found to be more vulnerable to the toxic effects of AgNPs with significant loss in mass which started at 50 mg kg^−1^ and AgNPs caused mortality at 125 mg kg^−1^ AgNPs. One possibility for this difference in toxicity is that *A. chlorotica* is a much smaller species than *A. caliginosa* with the mean mass of *A. chlorotica* at 200–300 mg, against 300–1200 mg for *A. caliginosa*. This could be the source of the difference in results and is supported by the fact that in this experiment, the smaller *A. caliginosa* died from exposure to higher levels of AgNPs (Table 1). Toxic effects vary between species; therefore, it is also possible that the discrepancy can be traced back to variations between *A. caliginosa* and *A. chlorotica* species.

#### Ag_2_S

Ag_2_S exposure did not cause death in *A. caliginosa* over the first 2 weeks of the experiment nor when the experiment was extended for a further 2 weeks. Neither mortality nor change in biomass was observed. This was different to the results obtained in the experiment examining acute toxicity of AgNPs where earthworms lost biomass over the course of two weeks. AgNPs also caused some mortality above 250 mg kg^−1^. This showed that the sulfidation of the AgNPs to Ag_2_S prevents their ability to release Ag^+^ ions due to their solubility constant, as Ag^+^ ions are reported to be a major factor in the toxicity of AgNPs (Shoults-Wilson et al. 2011b; Starnes et al. 2015). While pure silver readily reacts and releases reactive Ag^+^ ions, the bond between silver and sulphur is comparatively strong (Doolette et al. 2015) and Ag_2_S is less likely to react or release any ions. This reduced reactive behaviour causes a decline in toxic effects. In addition, the Ag_2_S particles used in this experiment were not nanoparticle size and therefore had a lower surface area to volume ratio.

### Reproduction

#### AgNP

Reprotoxic effects of AgNPs towards *A. caliginosa* at sublethal levels are limited. Due to the experimental set up using control groups, it can be assured that the decrease in cocoon production by earthworms exposed to 250 mg kg^−1^ AgNPs was not due to natural reproductive differences between individuals. This suggests that exposure to sublethal concentrations of AgNPs reduced the reproductive capacity in *A. caliginosa*, however, this effect was limited and may have been due to Ag^*+*^ becoming non-soluble over time by transformation compounds AgCl or Ag_2_S, or binding to organic matter which can reduce toxicity of AgNPs (Coutris et al. 2012). It also appears that *A. caliginosa* adapted to this new environment and regained their ability to produce cocoons with no long-term effects. In a study using *E. fetida*, AgNP concentrations of 727.6 mg kg^−1^ caused a significant decrease in reproduction (Shoults-Wilson et al. 2011a). In a 28-day study using *A. caliginosa*, Khalil (2016) found that AgNPs reduce the ability to produce cocoons starting at 100 mg kg^−1^ AgNPs, which is a lower threshold than in the experiments described here. However, 50 mg kg^−1^ AgNPs already caused a significant decrease in biomass during their study whereas exposure to 250 mg kg^−1^ AgNPs had no such effects here. The findings presented here are significantly different from those by Jesmer et al. (2017), who found a significant impact of AgNPs on the hatching rate of *Eisenia andrei* cocoons, while hatching rate was not affected in this study.

#### Ag_2_S

Neither cocoon production nor mean biomass showed any significant trends across treatments over the course of the experiment. This aligns with the hypothesis that Ag_2_S is less reactive than AgNPs and therefore less toxic, which was also shown in the acute toxicity experiment. In their paper on the fate of AgNPs during wastewater treatment, Ma et al. (2013) describe how AgNPs undergo a rapid transformation to Ag_2_S. The lower ability to cause toxicity can be explained by the low solubility of Ag_2_S and the associated inability to release Ag^+^ ions which would cause increased toxicity. When considering the ability of Ag_2_S to release ions, it helps to compare it with AgCl, of which only about 2 mg can be solved in water and has a Ksp ≈ 10^−9.75^ while Ag_2_S has a Ksp ≈ 10^-49^ (Choi et al. 2008). Therefore, the amount of potentially available Ag^+^ ions are very low in soil containing Ag_2_S.

### Avoidance

Avoidance is a more sensitive endpoint than acute toxicity experiments and can also be more sensitive than reproduction experiments. In a series of papers, *E. fetida* avoided soil containing as little as 10 mg kg^−1^ AgNPs while reproductive decline only occurred at levels above 700 mg kg^−1^ AgNPs (Shoults-Wilson et al. 2011c; 2011a). This was confirmed in this set of experiments as well, with linear gradient experiments using AgNPs and Ag_2_S both resulting in significant avoidance behaviour despite the lack of reproductive decline in the Ag_2_S reproduction experiment or the limited effects in the AgNP one. *Allolobophora chlorotica* avoid AgNPs at 12.5 mg kg^−1^ AgNPs (Brami et al. 2017), a further indication that this endogeic species is more sensitive towards AgNPs than *A. caliginosa*.

*Aporrectodea caliginosa* avoided soil containing Ag_2_S, regardless of the treatment. While acute toxicity tests did not show Ag_2_S to be directly toxic towards *A. caliginosa* and the reproductive study showed no long-term effects, *A. caliginosa* still appear to avoid it. This could be due to the shape of the particles being slightly abrasive towards the earthworms, so they avoid the area out of comfort rather than the substance being necessarily toxic.

It was noted that the biomass of the earthworms in this experiment declined from a mean of 686.72 mg before the experiment to 639.28 mg after the experiment. While not ideal, it was less than the 20%, which is generally accepted as the maximum mass earthworms should be allowed to lose during an experiment (Fründ et al. 2010).

## Conclusion

Overall, AgNPs and the resulting form, Ag_2_S, appear to be somewhat toxic towards *A. caliginosa* in all experiments. While AgNPs can be lethal towards *A. caliginosa* in large quantities, the reproduction experiment showed that earthworms can acclimatise to subtoxic conditions and continue to reproduce without permanent impact. So, if there were a contamination of soil with AgNPs, it appears that no lasting negative effect would persist on *A. caliginosa*, considering that 250 mg kg^−1^ is already a relatively high concentration of fresh particles to appear in the soil. It couldn’t be reached via the application of biosolids even if the AgNPs would not largely sulphidise, which would be expected to occur (Kaegi et al. 2013). However, a negative effect could be *A. caliginosa* avoiding or vacating the affected area, as seen in the linear gradient avoidance experiment.

Ag_2_S had no conclusive toxic effects on *A. caliginosa*. Observed avoidance behaviour did occur, but no negative impact was recorded on earthworm survival, biomass, cocoon production or hatchability of cocoons. This aligns with the widely held belief that the toxicity of AgNPs stems from their release of Ag^+^ ions (Shoults-Wilson et al. 2011b; Mcshan et al. 2014; Li et al. 2015). The reaction with sulphur to Ag_2_S creates a sufficiently strong bond to minimise or completely prevent the formation of Ag^+^ ions. Sulphidation of AgNPs lowers their antimicrobial potential, as shown by Reinsch et al. (2012) in a study with *Escherichia coli* and Schultz et al. (2018) similarly found Ag_2_S to have a limited antimicrobial effect. In another study, Levard et al. (2013) showed that even a low degree of sulphidation decreases the toxicity towards fish embryos, *C. elegans,* and duckweed.

However, while Ag_2_S does not negatively affect *A. caliginosa*, the avoidance behaviour could still cause a negative effect on the overall earthworm density of a soil if earthworms avoid the area. If the application of biosolids is a cause for the increase in Ag_2_S, it would probably be a widespread application leaving very little option for earthworms to avoid the area, which could potentially minimise the effects of any avoidance behaviour. In addition, the high organic matter content in biosolids can be a source of nutrition for the earthworms (Hartenstein and Neuhauser 1985; Pallant and Hilster 1996) which has the potential to reverse negative effects caused by Ag_2_S.

The data collected in this project is not sufficient to make definitive statements about the toxicity of Ag_2_S. The research community could usefully further investigate Ag_2_S toxicity in general, as it can be hypothesised that Ag_2_S presents no acute risk to the soil community. While other routes of silver entering the environment may still be of concern, the hazards of increased AgNPs in the wastewater system and subsequent application on to soil is less of a concern. Biosolids are already screened for specific metals (e.g. copper, cadmium, lead) which should not be applied in larger quantities (SI UK Statutory Intrument 1989), so addition of silver to this list is not necessary.

## Data Availability

Data available upon request.

## References

[CR1] Bart S, Amossé‚ J, Lowe CN, Mougin C, Péry AR, Pelosi C (2018). *Aporrectodea caliginosa*, a relevant earthworm species for a posteriori pesticide risk assessment: current knowledge and recommendations for culture and experimental design.. Environ Sci Pollut Res.

[CR2] Bouché, M 1977. Strategies lombriciennes. Ecol Bullet 122–132.

[CR3] Brami C, Glover AR, Butt KR, Lowe CN (2017). Effects of silver nanoparticles on survival, biomass change and avoidance behaviour of the endogeic earthworm *Allolobophora chlorotica*. Ecotoxicol Environ Safety.

[CR4] Choi JS, Park J-W (2015). Molecular characterization and toxicological effects of citrate-coated silver nanoparticles in a terrestrial invertebrate, the earthworm (*Eisenia fetida*). Mol Cell Toxicol.

[CR5] Choi O, Deng KK, Kim N-J, Ross L, Surampalli RY, Hu Z (2008). The inhibitory effects of silver nanoparticles, silver ions, and silver chloride colloids on microbial growth. Water Res.

[CR6] Coutris C, Joner EJ, Oughton DH (2012). Aging and soil organic matter content affect the fate of silver nanoparticles in soil. Sci Total Environ.

[CR7] DEFRA 2012. Waste water treatment in the United Kingdom – 2012 *In:* Department for environment, F. A. R. A. (ed.). www.defra.gov.uk

[CR8] Doolette CL, Mclaughlin MJ, Kirby JK, Batstone DJ, Harris HH, Ge H, Cornelis G (2013). Transformation of PVP coated silver nanoparticles in a simulated wastewater treatment process and the effect on microbial communities. Chem Central J.

[CR9] Doolette CL, Mclaughlin MJ, Kirby JK, Navarro DA (2015). Bioavailability of silver and silver sulfide nanoparticles to lettuce (*Lactuca sativa*): effect of agricultural amendments on plant uptake. J Hazard Mater.

[CR10] Fründ H-C, Butt K, Capowiez Y, Eisenhauer N, Emmerling C, Ernst G, Potthoff M, Schädler M, Schrader S (2010). Using earthworms as model organisms in the laboratory: recommendations for experimental implementations. Pedobiologia.

[CR11] Hartenstein R, Neuhauser E (1985). Stabilization of activated sludge: bioassay with *Eisenia fetida* and certain organic chemical characteristics. J Water Pollut Control Fed.

[CR12] Heckmann L-H, Hovgaard MB, Sutherland DS, Autrup H, Besenbacher F, Scott-Fordsmand JJ (2011). Limit-test toxicity screening of selected inorganic nanoparticles to the earthworm *Eisenia fetida*. Ecotoxicology.

[CR13] Jesmer AH, Velicogna JR, Schwertfeger DM, Scroggins RP, Princz JI (2017). The toxicity of silver to soil organisms exposed to silver nanoparticles and silver nitrate in biosolids-amended field soil. Environ Toxicol Chem.

[CR14] Jo Y, Kim BH, Jung G (2009). Antifungal activity of silver ions and nanoparticles on phytopathogenic fungi. Am Phytopathol Soc (APS).

[CR15] Kaegi R, Voegelin A, Ort C, Sinnet B, Thalmann B, Krismer J, Hagendorfer H, Elumelu M, Mueller E (2013). Fate and transformation of silver nanoparticles in urban wastewater systems. 47, 3866–3877. 10.1016/j.watres.2012.11.060 10.1016/j.watres.2012.11.060 23571111

[CR16] Khalil AM (2016). Physiological and genotoxic responses of the earthworm *Aporrectodea caliginosa* exposed to sublethal concentrations of AgNPs.. J Basic Appl Zool.

[CR17] Kim B, Park C-S, Murayama M, Hochella MF (2010). Discovery and characterization of silver sulfide nanoparticles in final sewage sludge products. Environ Sci Technol.

[CR18] Lamsa K, Kim S-W, Jung JH, Kim YS, Kim KS, Lee YS (2011). Inhibition effects of silver nanoparticles against powdery mildews on cucumber and pumpkin. Mycobiology.

[CR19] Levard C, Hotze EM, Colman BP, Dale AL, Truong L, Yang XY, Bone AJ, Brown GE, Tanguay Rl, Di Giulio RT, Bernhardt ES, Meyer JN, Wiesner MR, Lowry GV (2013). Sulfidation of silver nanoparticles: natural antidote to their toxicity. Environ Sci Technol.

[CR20] Li L, Wu H, Peijnenburg WJGM, Van Gestel CAM (2015). Both released silver ions and particulate Ag contribute to the toxicity of AgNPs to earthworm *Eisenia fetida*. Nanotoxicology.

[CR21] Lowe CN, Butt KR (2005). Culture techniques for soil dwelling earthworms: a review.. Pedobiologia.

[CR22] Lowe CN, Butt KR (2007). Earthworm culture, maintenance and species selection in chronic ecotoxicological studies: a critical review. Eur J Soil Biol.

[CR23] Lowe CN, Butt KR, Cheynier KY-M (2016). Assessment of avoidance behaviour by earthworms (*Lumbricus rubellus* and *Octolasion cyaneum*) in linear pollution gradients. Ecotoxicol Environ Safety.

[CR24] Ma R, Levard C, Judy JD, Unrine JM, Durenkamp M, Martin B, Jefferson B, Lowry GV (2013). Fate of zinc oxide and silver nanoparticles in a pilot wastewater treatment plant and in processed biosolids. Environ Sci Technol.

[CR25] Mcshan D, Ray PC, Yu H (2014). Molecular toxicity mechanism of nanosilver. J Food Drug Anal.

[CR26] Nowack B, Krug HF, Height M (2011). 120 years of nanosilver history: implications for policy makers. Environ Sci Technol.

[CR27] OECD (1984) Guideline for Testing of Chemicals “Earthworm, Acute Toxicity Tests” 207. Organisation for Economic Cooperation and Developement, Paris, France

[CR28] OECD (2016) Guideline for Testing of Chemicals “Earthworm Reproduction Test” (*Eisenia fetida*/*Eisenia andrei*) 222. Organisation for Economic Cooperation and Development, Paris, France

[CR29] Pallant E, Hilster LM (1996). Earthworm response to 10 weeks of incubation in a pot with acid mine spoil, sewage sludge, and lime. Biol Fertil Soils.

[CR30] Reinsch BC, Levard C, Li Z, Ma R, Wise A, Gregory KB, Brown GE, Lowry GV (2012). Sulfidation of silver nanoparticles decreases *Escherichia coli* growth inhibition.. Environ Sci Technol.

[CR31] Sadovnikov SI, Kuznetsova YV, Rempel AA (2016). Ag_2_S silver sulfide nanoparticles and colloidal solutions: synthesis and properties. Nano-Struct Nano-Object.

[CR32] Schultz CL, Gray J, Verweij RA, Busquets-Fité M, Puntes V, Svendsen C, Lahive E, Matzke M (2018). Aging reduces the toxicity of pristine but not sulphidised silver nanoparticles to soil bacteria. Environ Sci Nano.

[CR33] Shoults-Wilson WA, Reinsch BC, Tsyusko OV, Bertsch PM, Lowry GV, Unrine JM (2011). Effect of silver nanoparticle surface coating on bioaccumulation and reproductive toxicity in earthworms (*Eisenia fetida*). Nanotoxicology.

[CR34] Shoults-Wilson WA, Reinsch BC, Tsyusko OV, Bertsch PM, Lowry GV, Unrine JM (2011). Role of particle size and soil type in toxicity of silver nanoparticles to earthworms. Soil Sci Soc Am J.

[CR35] Shoults-Wilson WA, Zhurbich OI, Mcnear DH, Tsyusko OV, Bertsch PM, Unrine JM (2011). Evidence for avoidance of Ag nanoparticles by earthworms (*Eisenia fetida*). Ecotoxicology.

[CR36] SI UK Statutory intrument (1989) The Sludge (Use in Agriculture) Regulations 1989. Statutory Instrument No. 1263. HMSO, London

[CR37] Sims RW, Gerard BM (1999) Earthworms: keys and notes for the identification and study of the species, Brill Archive

[CR38] Starnes DL, Unrine JM, Starnes CP, Collin BE, Oostveen EK, Ma R, Lowry GV, Bertsch PM, Tsyusko OV (2015). Impact of sulfidation on the bioavailability and toxicity of silver nanoparticles to *Caenorhabditis elegans*. Environ Pollut.

